# Factors affecting the duration of coronary artery lesions in patients with the Kawasaki disease: a retrospective cohort study

**DOI:** 10.1186/s12969-021-00589-z

**Published:** 2021-06-26

**Authors:** Xuting Zhang, Yuee He, Yiping Shao, Biyao Hang, Zhipeng Xu, Maoping Chu

**Affiliations:** Children’s Heart Center, The Second Affiliated Hospital and Yuying Children’s Hospital, Institute of Cardiovascular Development and Translational Medicine, Wenzhou Medical University, Wenzhou, 325000 Zhejiang, China

**Keywords:** Kawasaki disease, Mucocutaneous lymph node syndrome, Prognosis, Survival analysis, Coronary artery lesions, Kaplan–Meier curve, Multivariate cox regression model

## Abstract

**Background:**

Coronary artery lesions (CALs) are the most severe complication of Kawasaki disease (KD). Approximately 9–20% of the patients with KD develop CAL despite receiving regular treatment (intravenous immunoglobulin [IVIG] and aspirin). Some patients develop coronary aneurysms, leading to coronary artery stenosis or thrombosis, resulting in ischaemic heart disease and significantly affect the patients’ lives. The purpose of this study was to investigate the factors associated with the duration of CAL in patients with KD.

**Methods:**

The data of 464 patients with KD and CAL admitted to the Children’s Heart Centre, The Second Affiliated Hospital and Yuying Children’s Hospital from 2010 to 2018 were retrospectively analysed. Demographic and clinical information and echocardiographic follow-up data were collected. Kaplan–Meier curves were used to estimate the overall CAL duration, and the log-rank test was used to compare statistical differences. Univariate and multivariate Cox regression models were used to identify variables related to the CAL duration.

**Results:**

The median CAL duration was 46 days (95% confidence interval: 41–54 days). CALs were observed in 61.5, 41.5, 33.3, 22.3, 10.3, and 7.7% of the patients at 1 month, 2 months, 3 months, 6 months, 1 year, and 2 years after the onset of KD, respectively. Univariate Cox regression model showed that sex (*p* = 0.016), rash symptoms (*p* = 0.035), delayed IVIG treatment (*p* = 0.022), CAL type (*p* < 0.001), degree of CAL (*p* < 0.001), white blood cell count before IVIG treatment (*p* = 0.019), and platelet count after IVIG treatment (*p* = 0.003) were statistically significant factors associated with the overall CAL duration. Multivariable Cox regression showed that delayed IVIG treatment (*p* = 0.020), multiple dilatations (*p* < 0.001), a greater degree of dilatation (*p* < 0.001), and higher platelet count after IVIG treatment (*p* = 0.007) were positively related to CAL duration.

**Conclusions:**

CAL duration was affected by delayed IVIG treatment, type of CAL, degree of CAL, and platelet count after IVIG treatment. These factors should be monitored carefully during the follow-up and management of patients with KD and CAL.

## Background

Kawasaki disease (KD) is an acute febrile vasculitis of unknown aetiology that predominantly affects children aged < 5 years. Its pathological features include systemic inflammation of medium-sized blood vessels and various tissues and organs. The coronary arteries are typically the most affected vessels [1, 2]. Although KD is generally considered self-limiting and treated with high-dose intravenous immunoglobulin (IVIG), 9–20% of the patients with KD develop coronary artery lesions (CALs) and 4% develop coronary artery aneurysms (CAAs) [3–6].

Persistent CALs may lead to thrombosis, stenosis, and obstruction, causing major adverse cardiac events, such as unstable angina pectoris and myocardial infarction, and even death [7, 8]. One study reported that 5% of the acute coronary syndromes in patients aged < 40 years with suspected myocardial ischaemia were related to CAA secondary to KD [9]. Several previous studies have reported that patients with more severe CAL, especially those with aneurysms, tend to have a longer CAL duration and a greater risk of developing coronary events later in life [10, 11]. Thus, several guidelines for patients with CAL recommend follow-up plans according to the degree of CAL [1, 2].

Recently, paediatricians have focused on patients with KD and CAA [12–16]. However, patients with coronary artery dilation, but not CAA, comprise the majority of the patients with CAL [3, 5]. Although coronary artery dilation in most patients normalizes within 2 months after its onset, CAL persists or progresses in some patients [1, 2, 11]. Considering the large number of patients affected by KD worldwide, these patients cannot be ignored. In addition to the degree of CAL, previous studies have reported several other factors related to the prognosis of patients with KD with CAL; however, no consensus has been achieved regarding these factors [10, 11, 17, 18]. Therefore, we conducted a retrospective cohort study to determine the prognosis of all patients with KD with CAL and determine the relationship between factors, such as laboratory data, treatment plans, IVIG resistance, and CAL duration.

## Methods

### Patients

**Fig. 1:**
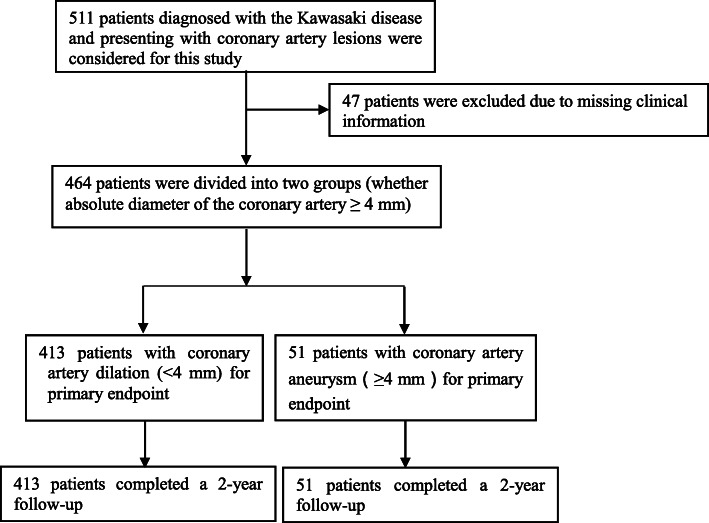
Patient flow chart. Flow chart showing the demographic and clinical information of all study participants. A total of 47 patients were excluded; 464 patients were included in the final analysis. The patients were divided into two groups based on the degree of coronary artery lesions

We collected the demographic and clinical information of 511 patients with KD and CAL admitted to the Children’s Heart Centre of The Second Affiliated Hospital and Yuying Children’s Hospital from 1 January 2010 to 31 December 2018. KD diagnosis was based on the 2017 American Heart Association (AHA) guidelines and CAL diagnosis was based on the expert consensus in this region. Due to missing clinical information, 47 patients were excluded; therefore, 464 patients were included in the final analysis. Patients were divided into two groups based on the degree of CAL (patients with multiple CALs were grouped according CAL severity): the dilation group (absolute diameter of the coronary artery < 4 mm) and the aneurysm group (absolute diameter of the coronary artery ≥4 mm) (Fig. 1).

### Data collection

The patients’ medical data and follow-up records, including the patients’ age, weight, sex, KD type, clinical symptoms, IVIG resistance, delayed IVIG treatment, CAL type, and degree of CAL, were collected from the electronic medical record system of the hospital. Laboratory data including the white blood cell, neutrophil, and platelet counts (10^9^/L); haematocrit (%); erythrocyte sedimentation rate (mm/h); and haemoglobin (g/L), C-reactive protein (mg/L), alanine aminotransferase and aspartate aminotransferase (U/L), albumin (g/L), sodium (mmol/L), potassium (mmol/L), chloride (mmol/L), and brain natriuretic peptide (pg/mL) levels were recorded before and after IVIG treatment.

Patients underwent two or three echocardiography during their hospitalization, often with an interval of 4–5 days. The follow-up echocardiography was conducted once every 2 weeks within 6 months after discharge and once every 1–2 months in the later period. Every echocardiography was performed by at least two experienced paediatric echocardiographers. For the echocardiographic follow-up of the same patient, our heart centre used the same echocardiographers as much as possible. The absolute dimensions of the left main coronary artery (LMCA), left anterior descending artery (LAD), left circumflex (LCX), right coronary artery (RCA), and posterior descending coronary artery (PDCA) were recorded each time. This study’s primary endpoint was the overall duration of CAL, defined as the duration between the first date of CAL detection on echocardiography and the date of its disappearance on echocardiography. The disappearance of CAL in echocardiography was defined as normalization of absolute inner diameter, which means the absolute inner diameter is smaller than the diagnostic criteria.

### Diagnosis of KD

According to the 2017 AHA guidelines, the clinical criteria for the diagnosis of KD include a prolonged, unexplained fever lasting for ≥5 days and the presence of four or more of the five major clinical symptoms, namely bilateral conjunctival congestion, polymorphous rash, oral changes, acute non-purulent cervical lymphadenopathy, and extremity changes [1].

A patient with unexplained fever for > 5 days, < 4 of the major clinical symptoms, and supportive laboratory findings or coronary artery abnormalities was considered to have incomplete (or atypical) KD [1].

IVIG resistance was defined as a body temperature of > 38.5 °C at 48 h after the end of IVIG infusion [19]. Delayed IVIG treatment was defined as the administration of IVIG > 10 days after disease onset [1, 2].

CALs were classified according to their size as follows: absolute coronary artery diameter > 2.5 mm for children aged < 3 years, > 3 mm in children aged 3–9 years, and > 3.5 mm in children aged > 9 years. CAAs were diagnosed if the absolute diameter of the coronary artery was ≥4 mm. The range of small, medium, and giant CAAs was defined by a diameter < 5, 5–8, and > 8 mm, respectively [20]. Single CAL was defined as CAL in one of the following arteries: LMCA LAD, LCX, RCA, or PDCA. Multiple CAL was defined as CAL in more than one of these arteries.

### Statistical methods

Continuous variables are expressed as means ± standard deviation or interquartile range, and categorical variables are summarised as numbers and percentages. Continuous and categorical variables were compared among the groups using the *t*- or the Mann–Whitney’s *U* tests (as appropriate) and the Pearson’s Chi-square or the Fisher’s exact tests (as appropriate), respectively. The overall duration of CAL was estimated using the Kaplan–Meier curve method, and the log-rank test was used to compare the variables. A univariate Cox analysis was conducted to explore the variables associated with the overall duration of CAL. Statistically significant variables in the univariate Cox regression analysis were included in the multivariate Cox model. Statistical significance was set at *p* < 0.05. All statistical analyses were performed using the R software (version 3.6.1, R Foundation for Statistical Computing, Vienna, Austria). The Cox regression analyses were performed using the ‘survival’ package and the Kaplan–Meier curves were created using the R package ‘ggplot2’.

## Results

### Demographic and clinical characteristics

Of the 464 patients with KD included in this study, 411 and 53 were categorised into the dilation and aneurysm groups, respectively. Patients in the aneurysm group were significantly older (*p* < 0.001) and heavier (*p* = 0.002) than those in the dilation group (Table 1). The proportions of patients with rash (*p* = 0.007) and oral symptoms (*p* = 0.025) in the aneurysm group were lower than those in the dilation group. A higher proportion of patients in the aneurysm group received delayed IVIG treatment (*p* = 0.018). In addition, more patients in the aneurysm group had multiple CALs (*p* < 0.001) than the dilation group. There were no significant differences in the sex, KD type, other clinical symptoms, or IVIG resistance between the two groups (Table 1).

**Table 1:** Patient characteristics

	Dilation group	Aneurysm group	*P* value
	*N* = 413 (89.0%)	*N* = 51 (11.0%)	
Age (months)	18.3 (10.6–28.4)	35.7 (15.1–63.8)	<0.001^*^
Weight (kg)	11.0 (9.4–13.0)	14.0 (11.0–20.0)	0.002^*^
Sex (M/F)	308/105	43/8	0.175
KD type			0.799
Complete	321 (77.7%)	41 (80.4%)	
Incomplete	92 (22.3%)	10 (19.6%)	
Symptoms			
Fever	410 (99.3%)	51 (100%)	0.999
LN enlargement	181 (43.8%)	22 (43.1%)	0.999
Rash	312 (75.5%)	29 (56.9%)	0.007^*^
Conjunctivitis	377 (91.3%)	48 (94.1%)	0.674
Oral changes	366 (88.6%)	39 (76.5%)	0.025^*^
Extremity changes	287 (69.5%)	32 (62.7%)	0.412
Delayed IVIG treatment	45 (10.9%)	12(23.5%)	0.018^*^
IVIG-resistant	32 (7.7%)	7 (13.7%)	0.236
CAL types			<0.001^*^
Single	32 7 (79.2%)	15 (29.4%)	
Multiple	86 (20.8%)	36 (70.6%)	

Continuous data are shown as median (interquartile range). Categorical data are shown as number (percentage)

Abbreviations: *CAL* coronary artery lesion; *M* male; *F* female; *KD* Kawasaki disease; *IVIG* intravenous immunoglobulin; *LN* lymph node

Delayed IVIG was defined as the administration of IVIG > 10 days after disease onset

Single CAL was defined as CAL in one of the following arteries: LMCA LAD, LCX, RCA, or PDCA.. Multiple CAL was defined as CAL in more than one of these arteries

The neutrophil count was higher (*p* = 0.027), and the aspartate aminotransferase level was lower in the aneurysm group (*p* = 0.029) than in the dilation group before IVIG treatment. There were no significant differences in the laboratory data after IVIG treatment between the two groups. The detailed demographic and clinical characteristics are presented in Tables 2 and 3.

**Table 2:** Laboratory data before the initiation of IVIG treatment

	Dilation group (*N* = 413)	Aneurysm group (*N* = 51)	*P* value
White blood count (10^9^/L)	15.3 (11.8–19.3)	16.6 (13.0–21.0)	0.281
Haemoglobin (g/L)	110.3 ± 10.9	111.4 ± 10.1	0.482
Haematocrit (%)	0.33 (0.32–0.36)	0.33 (0.31–0.36)	0.365
Platelet count (10^9^/L)	393.7 ± 159.7	411.9 ± 159.9	0.447
Neutrophil count (10^9^/L)	10.4 ± 5.2	12.0 ± 4.9	0.027^*^
C-reactive protein (mg/L)	76.0 (43.0–119.0)	88.0 (51.9–136.0)	0.141
Erythrocyte sedimentation rate (mm/h)	35.2 ± 13.9	37.5 ± 12.9	0.235
Alanine aminotransferase (U/L)	39.0 (20.0–93.0)	35.0 (18.5–65.0)	0.064
Aspartate aminotransferase (U/L)	33.0 (25.0–52.0)	27.0 (23.0–43.5)	0.029^*^
Albumin (g/L)	36.9 ± 5.8	35.6 ± 5.2	0.107
Sodium (mmol/L)	135.7 ± 2.5	135.8 ± 2.4	0.728
Potassium (mmol/L)	4.37 ± 0.53	4.23 ± 0.57	0.098
Chloride (mmol/L)	101.7 ± 3.1	101.0 ± 2.9	0.125
Brain natriuretic peptide (pg/mL)	887 (395–2213)	656 (195.5–1865)	0.539

Data are shown as mean ± standard deviation or median (interquartile range)

Abbreviation: *IVIG* intravenous immunoglobulin

**Table 3:** Laboratory data after IVIG treatment

	Dilation group (*N* = 413)	Aneurysm group (*N* = 51)	*P* value
White blood count (10^9^/L)	8.4 (6.6–10.6)	8.0 (5.4–10.9)	0.741
Haemoglobin (g/L)	107.3 ± 10.9	109.3 ± 11.9	0.258
Haematocrit (%)	0.33 (0.31–0.35)	0.33 (0.31–0.35)	0.474
Platelet count (10^9^/L)	555.0 ± 189.7	517.1 ± 173.5	0.150
Neutrophil count (10^9^/L)	3.3 ± 2.3	4.7 ± 6.5	0.132
C-reactive protein (mg/L)	8.7 (5.0–16.0)	8.0 (4.0–17.4)	0.364
Alanine aminotransferase (U/L)	29.0 (20.0–48.0)	34.0 (23.5–505.5)	0.730
Aspartate aminotransferase (U/L)	46.0 (38.0–61.0)	45.0 (37.5–66.5)	0.418
Albumin (g/L)	35.6 ± 4.0	34.9 ± 5.2	0.338
Brain natriuretic peptide (pg/mL)	231 (159–336)	278 (201–441)	0.143

Data are shown as mean ± standard deviation or median (interquartile range)

Abbreviation: *IVIG* intravenous immunoglobulin

### Kaplan–Meier curve and cox proportional hazards regression analyses

**Fig. 2:**
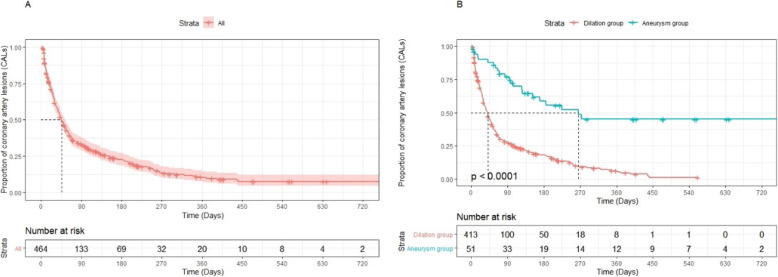
Kaplan–Meier curves for coronary artery lesion duration. **A**. The duration of coronary artery lesions (CALs) for all patients is shown. The median CAL duration was 46 days (95% confidence interval [CI]: 41–54 days). **B**. The durations of coronary artery lesions for patients with dilation (absolute diameter of coronary artery < 4 mm) and aneurysm (absolute diameter of coronary artery ≥ 4 mm) are shown. The CAL duration was prolonged once the KD patients developed CAA (41 days vs. 265 days)

**Fig. 3:**
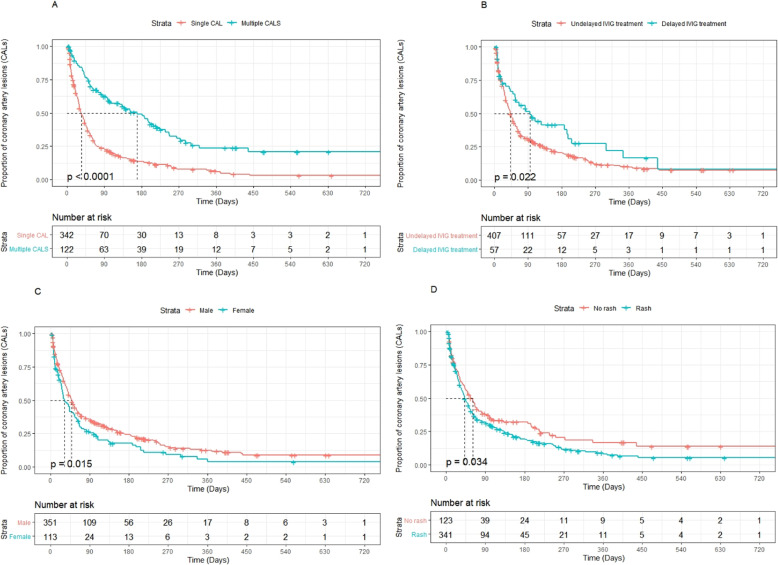
Kaplan–Meier curves for coronary artery lesion duration paired with independent variables. **A**. The duration of coronary artery lesions (CALs) in terms of the type of CAL is shown. Patients with multiple CALs had a statistically longer period of CAL than those with single CAL. **B**. The duration of CALs in terms of the timing of intravenous immunoglobulin (IVIG) treatment is shown. Patients who received delayed IVIG treatment had a statistically longer duration of CAL than those who did not. **C**. The duration of CALs in terms of sex is shown. Male patients had a statistically longer duration of CAL than female patients. **D**. The duration of CALs in terms of the presence of a rash is shown. Patients with rash symptoms had a statistically longer duration than those without such symptoms

The median CAL duration was 46 days (95% confidence interval [CI]: 41–54 days). CALs were observed in 61.5, 41.5, 33.3, 22.3, 10.3, and 7.7% of the patients at 1 month, 2 months, 3 months, 6 months, 1 year, and 2 years after disease onset, respectively (Fig. 2A). The median CAL duration in the dilation and aneurysm groups was 41 days (95% CI: 35–46 days) and 265 days (95% CI: 152–720 days), respectively (Fig. 2B). As shown in Fig. 3, the CAL duration was prolonged once the KD patients developed CAA.

The results of the univariate Cox regression analysis revealed that the CAL type (single vs. multiple) (p<0.001), delayed IVIG treatment (*p* = 0.003), sex (*p* = 0.016), and rash symptoms (*p* = 0.035) were correlated with the CAL duration (Fig. 3A-D). The degree of CAL (p<0.001, Fig. 2B), white blood cell count before IVIG therapy (*p* = 0.019), and platelet count after IVIG therapy (p = 0.003) were also associated with the CAL duration (Table 4). Delayed IVIG treatment (hazard ratio [HR]: 0.722; 95% CI: 0.550–0.950; *p* = 0.020), multiple dilations (HR: 0.516; 95% CI: 0.392–0.677; *p* < 0.001), greater dilation (HR: 0.319; 95% CI: 0.202–0.505; *p* < 0.001), and increased platelet count after IVIG (HR: 0.999; 95% CI: 0.9986–0.9998; *p* = 0.007) were found to be independently associated with the CAL duration (Table 4).

**Table 4:** Factors associated with CAL duration

	Univariate		Multivariate	
	HR (95% CI)	*P* value	HR(95% CI)	*P* value
Sex				
Male	Reference	1.00	Reference	1.00
Female	1.334 (1.055–1.686)	0.016^*^	1.215 (0.958–1.540)	0.108
Rash				
Yes	Reference	1.00	Reference	1.00
No	1.295 (1.019–1.645)	0.035^*^	0.976 (0.761–1.251)	0.846
IVIG treatment				
Not delayed	Reference	1.00	Reference	1.00
Delayed	0.672 (0.515–0.875)	0.022^*^	0.722 (0.550–0.950)	0.020^*^
CAL type				
Single	Reference	1.00	Reference	1.00
Multiple	0.382 (0.294–0.496)	<0.001^*^	0.516 (0.392–0.677)	<0.001^*^
Degree of CAL				
Only dilation	Reference	1.00	Reference	1.00
Aneurysm	0.246 (0.158–0.382)	<0.001^*^	0.319 (0.202–0.505)	<0.001^*^
Laboratory data				
White blood cell count before IVIG	0.979 (0.962–0.997)	0.019	1.004 (0.981–1.020)	0.971
Platelet count after IVIG	0.999 (0.998–0.999)	0.003	0.9992 (0.9986–0.9998)	0.007^*^

Abbreviations: *CAL* coronary artery lesion; *IVIG* intravenous immunoglobulin; *HR* hazard ratio

Delayed IVIG was defined as the administration of IVIG > 10 days after disease onset

Single CAL was defined as CAL in one of the following arteries: LMCA LAD, LCX, RCA, or PDCA. Multiple CAL was defined as CAL in more than one of these arteries

Dilation was defined as an absolute diameter of the coronary artery of < 4 mm

Aneurysm was defined as an absolute diameter of the coronary artery of ≥4 mm

## Discussion

To our knowledge, the present investigation is one of the largest studies to focus on the prognosis of patients with KD and CAL and explore the risk factors associated with CAL duration. Over the two-year follow-up, the CALs resolved in 312/464 patients, and the median regression time was 46.0 days. The regression rate of 67.2% in this study is lower than that reported in previous studies [10, 11, 21].

We found that the degree of CAL was associated with the prognosis, which is consistent with the previously reported findings. Miura et al. reported that the greater the coronary dilation, the higher the probability of future adverse cardiovascular events [7]. Other studies have reported that small and medium CAAs resolve more frequently than giant CAAs, which is consistent with our findings [11, 14, 22]. KD can lead to severe coronary artery damage. We found that the duration of CALs in children with multiple CALs was longer than that in children with a single CAL. The severity of CAL reflects the extent of inflammation. The following three linked KD vasculopathy process primarily involve muscular arteries: necrotising arteritis, subacute/chronic vasculitis, and luminal myofibroblastic proliferation (LMP) [1]. Orenstein et al. reported that subacute/chronic vasculitis may occur or persist for several months or years and that LMP causes progressive coronary arterial stenosis and thrombosis [8], which occur more frequently in patients with CAA.

Paediatricians often monitor the platelet count in patients with KD. In Japan, the platelet count is used to predict IVIG-resistant coronary aneurysms [23, 24]. The natural trend of the platelet count in patients with KD can be divided into three different stages. First, the platelet count is normal, and no platelet aggregation is seen. Then, the platelets are activated, and the platelet count increases. This typically occurs as fever improves, and the rash begins to scale, often in the second or third week of the disease. The changes at this stage are due to the release of serotonin from the platelets. Finally, the platelet count returns within the normal range; however, children with coronary aneurysms continue to be at a risk of elevated platelet counts [25, 26]. Therefore, during the early stage of KD, the platelet count decreases before it increases slowly. Patients with higher platelet counts (> 450 × 10^9^/L) are more likely to develop CAA and not respond to IVIG than patients with normal platelet counts (150–450 × 10^9^/L) [25]. In our study, the platelet count measured before IVIG treatment initiation was lower in the aneurysm group than in the dilation group. The platelet count measured after IVIG treatment was higher in the aneurysm group than in the dilation group, although these differences were not significant. We found that the longer the duration of coronary artery dilation, the higher the platelet count after IVIG, which is consistent with previous studies.

According to several guidelines, the optimal time for administering IVIG is 5–10 days after the onset of illness [1, 2]. The late onset of typical symptoms in some patients with KD or difficulty in referring patients to specialists may delay the IVIG treatment. Previous studies [27, 28] have reported that delayed IVIG treatment may lead to an increased risk of CAL and longer CAL durations in patients with KD, which are consistent with our results.

We did not find a relationship between IVIG resistance and the duration of CALs. This may be due to repeated IVIG treatments or additional corticosteroid treatments in patients who did not initially respond to the treatment, resulting in a suppressed immune response and inflammation. Therefore, no difference in the immune function was observed between patients who received multiple rounds of IVIG or additional corticosteroid treatment and patients who received only one round of IVIG. We did not find any other significant associations between other clinical symptoms or laboratory data and the duration of CALs.

This study is not without limitations. First, because CAL was detected by echocardiography, both the occurrence and disappearance of CAL might show earlier than the check time point. According to previous research, the occurrence and the disappearance of CAL mostly happen in the subacute phase. Therefore, the follow-up strategy was made because the subacute-phase follow-up examination was more intensive than the chronic phase. We tried to make the duration of CAL obtained from the echocardiography closer to the real-time. Second, as this study began in 2010, we did not use the z-score to determine the degree of CALs, which has recently been reported as more appropriate [21, 29]. Instead, we used the previous standard measurement of absolute luminal diameter to assess the severity of CAL. This resulted in the aneurysm group being significantly older than the dilation group. Third, the data may have been biased as they were obtained from a clinical centre. Fourth, the follow-up period of 2 years was not long enough to determine the outcome of all patients with CAL.

## Conclusions

In this retrospective study, the duration of CAL was longer in patients with KD with a greater degree of CAL, multiple CALs, higher platelet counts after IVIG, and delayed IVIG treatment. Therefore, these factors should be monitored carefully by paediatricians. Cardiac coronary artery examinations, including angiography and percutaneous coronary interventions, and anticoagulant therapies should be used in patients with KD.

## Data Availability

The datasets generated during and analysed during the current study are not publicly available due to data protection but are available from the corresponding author on reasonable request.

## Abbreviations

KD: Kawasaki disease
IVIG: Intravenous immunoglobulin
CAL: Coronary artery lesion
CAA: Coronary artery aneurysm
AHA: American heart association
LMCA: Left main coronary artery
LADA: Left anterior descending artery
LCX: Left circumflex
RCA: Right coronary artery
PDCA: Posterior descending coronary artery
LMP: Luminal myofibroblastic proliferation
CI: Confidence interval
HR: Hazard ratio
M: Male
F: Female
LN: Lymph node
